# Selective utilization of non‐homologous end‐joining and homologous recombination for DNA repair during meiotic maturation in mouse oocytes

**DOI:** 10.1111/cpr.13384

**Published:** 2022-12-23

**Authors:** Crystal Lee, Jiyeon Leem, Jeong Su Oh

**Affiliations:** ^1^ Department of Integrative Biotechnology Sungkyunkwan University Suwon South Korea; ^2^ Biomedical Institute for Convergence at SKKU (BICS) Sungkyunkwan University Suwon South Korea

## Abstract

DNA double‐strand breaks (DSBs) are highly toxic lesions that can cause genomic instability and can be repaired by non‐homologous end‐joining (NHEJ) and homologous recombination (HR) pathways. Despite extensive studies about DSB repair pathways, the roles of each pathway during meiotic maturation in oocytes are not well understood. Here we show that oocytes selectively utilize NHEJ and HR to repair DSBs during meiotic maturation. Inhibition of NHEJ impaired the meiotic maturation of oocytes with DNA damage by activating the spindle assembly checkpoint (SAC) with a concomitant increase in metaphase I (MI) arrest and DNA damage levels. In contrast, oocytes with DNA damage bypassed SAC‐mediated MI arrest despite the presence of fragmented DNA when HR was inhibited. Notably, this bypass of SAC arrest by HR inhibition was associated with a loss of centromere integrity and subsequent impairment of chromosome architecture. Our results demonstrate that, while NHEJ is critical for the meiotic maturation of oocytes with DNA damage, HR is essential to maintain centromere integrity against DNA damage during meiotic maturation, revealing distinct roles of NHEJ and HR during meiotic maturation in mouse oocytes.

## Graphical abstract

In oocytes with DNA damage, inhibition of NHEJ impairs meiotic maturation by increasing DNA damage levels and, subsequently, the incidence of SAC‐mediated MI arrest. In contrast, inhibition of HR decreases centromere integrity, which in turn causes DNA fragmentation during chromosome segregation.

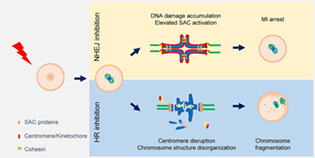

## 1 INTRODUCTION

DNA double‐strand breaks (DSBs) are one of the most deleterious types of DNA damage and often occur naturally through various endogenous and exogenous processes. However, cells are able to cope with DSBs using well‐equipped repair pathways to maintain genome integrity. DSBs are primarily repaired by two mechanisms: non‐homologous end‐joining (NHEJ) and homologous recombination (HR).^1^ NHEJ is initiated by the recognition and binding of the Ku70/80 heterodimer to DNA ends, followed by recruitment of the main NHEJ repair factors, including DNA‐dependent protein kinase catalytic subunit (DNA‐PKcs), Artemis and DNA ligase IV.^2^ Because DSB ends are directly ligated without the use of a homologous template, NHEJ is generally considered error‐prone. By contrast, HR tends to be error‐free because broken ends are repaired using homologous sequences as templates. Also, HR is a more complex process involving 5′‐end resections to generate 3′‐single‐stranded (ss) DNA overhangs.^3^ This processing is initiated by the binding of the heterotrimeric Mre11‐Rad50‐Nbs1 (MRN) complex to DSBs, which coordinates tethering and short‐range nucleolytic degradation of DSB ends by activating the endo‐ and exonuclease activity of Mre11. After 5′‐end‐resection, the 3’‐ssDNA overhangs are rapidly coated by RPA, which is subsequently displaced by Rad51, forming presynaptic filaments that facilitate the search for homologous DNA templates for strand invasion and repair.^4^ The choice between these two major pathways for DSB repair is linked to the progression of the cell cycle. Since NHEJ does not require a homologous template, it is active throughout all phases of the cell cycle but represents the major pathway in G1 phase. On the other hand, the need for extensive homology in HR restricts this mechanism to the S and G2 phases of the cell cycle when following DNA replication.^5^

Mammalian oocytes are arrested in the prophase of the first meiosis for several months or decades, depending on the species.^6^ During this prolonged time, they may be subjected to ongoing exogenous and endogenous assaults. Therefore, oocytes appear to be particularly susceptible to accumulating DNA damage. Surprisingly, however, oocytes are deficient in a robust G2/M DNA damage checkpoint, unlike somatic cells.^7^, ^8^, ^9^, ^10^ Thus, oocytes with DNA damage resume meiosis and undergo germinal vesicle (GV) breakdown (GVBD) when the damage is not severe. However, oocytes with DNA damage are arrested at the metaphase of the first meiosis (MI) during meiotic maturation.^9^, ^10^ Notably, DNA damage‐induced MI arrest is independent of ATM/ATR but caused by the activation of the spindle assembly checkpoint (SAC).^9^, ^10^ Interestingly, this DNA damage‐induced SAC arrest is not associated with aberrant kinetochore‐microtubule (kMT) attachments,^11^ implying that oocytes have a unique DNA damage response (DDR). Although the DNA damage response in oocytes differs from that in somatic cells, there are strong indications that DNA repair occurs in oocytes. Indeed, recent studies have shown that oocytes are able to repair DSBs during prophase arrest, indicating that oocytes are equipped with DDR machineries and have the capacity to repair damaged DNA.^8^, ^9^, ^12^ Moreover, metaphase II (MII) oocytes have been shown to repair DSBs using NHEJ.^13^ Consistent with this, transcriptome analyses demonstrated that components of both HR and NHEJ are expressed in mammalian oocytes.^13^, ^14^, ^15^ However, it remains unclear how oocytes utilize HR and NHEJ repair pathways in response to DNA damage during meiotic maturation.

In the present study, we investigate the roles of the two main repair pathways, HR and NHEJ, during meiotic maturation in mouse oocytes. We found that HR is predominant in GV‐arrested oocytes, whereas NHEJ is preferred in matured MII oocytes. Inhibition of NHEJ caused SAC‐mediated MI arrest in oocytes with DNA damage. In contrast, inhibition of HR allowed oocytes with DNA damage to bypass SAC‐mediated MI arrest despite the presence of fragmented DNA. This bypass is associated with a loss of centromere integrity and subsequently impaired chromosome architecture. Therefore, our results suggest that oocytes utilize both HR and NHEJ pathways to repair DSBs during meiosis, with each pathway having a distinctive role and its dominancy varies preferentially along the different stages of maturation.

## 2 MATERIALS AND METHODS

### 2.1 Oocyte collection and culture

Female three‐week‐old ICR mice (Koatech, South Korea) were used in all experiments. Experiments were approved by the Institutional Animal Care and Use Committees of Sungkyunkwan University (ID: SKKUIACUC2021‐08‐23‐1). Oocytes were isolated from the ovaries of female mice primed with 5 IU pregnant mare serum gonadotropin (PMSG) 46–48 h before collection. Oocytes were cultured in M2 medium supplemented with 100 μM 3‐isobutyl‐1‐methylxanthine (IBMX) to prevent meiotic resumption. Regarding meiotic maturation, oocytes were cultured in IBMX‐free M2 medium under mineral oil at 37°C in a 5% CO_2_ incubator. Oocytes were then imaged every 1 h for up to 16 h on a Nikon Eclipse Ti inverted microscope by checking for the disappearance of the GV and appearance of polar body for analysis of GVBD and polar body extrusion (PBE), respectively.

Regarding chemical treatment, oocytes were treated with 50 μg/ml etoposide (ETP), 50 μM B02 (Tocris), 20 μM SCR7, or 2 μM AZ3146 (Selleck Chemicals). All chemicals were dissolved in dimethyl sulfoxide (DMSO) and used at a dilution of 0.1% or below. Chemicals and culture media were purchased from Sigma‐Aldrich unless stated otherwise.

### 2.2 Time‐lapse imaging of oocytes

Time‐lapse imaging was performed using a Nikon Eclipse Ti inverted microscope equipped with a CCD cooled camera (DS‐Qi1Mc, Nikon). To visualize spindle microtubules and chromosomes, we added SiR‐tubulin and SiR‐DNA (Cytoskeleton, Inc.) to the culture medium at a final concentration of 100 nM, as validated previously in mouse oocytes.^16^, ^17^

### 2.3 Immunostaining

Oocytes were fixed in 4% paraformaldehyde (PFA) for 20 mins and permeabilized in PBS with 0.1% Triton X‐100 and 0.01% Tween 20 for 30 mins. After permeabilization, oocytes were blocked in 3% bovine serum albumin (BSA) in PBS for 1 h at room temperature. Oocytes were incubated overnight at 4°C with primary antibodies and then for 2 h at room temperature with secondary antibodies. Chromosomes were counterstained with DAPI. Oocytes were examined under an LSM 900 confocal laser scanning microscope (Zeiss).

Oocytes were exposed to ice‐cold M2 media for 10 mins and immediately subjected to immunostaining as described above to analyse kMT attachment.

### 2.4 Antibodies

The following primary antibodies were used: anti‐γ‐H2AX (Abcam, ab22551, 1:250), anti‐MDC1 (Abcam, ab241048, 1:100), anti‐acetylated‐α‐tubulin (Sigma‐Aldrich, T7451, 1:500; Abcam, ab179484, 1:500), anti‐Mad2 (Abcam, ab70383, 1:100), anti‐BubR1 (Abcam, ab28193, 1:100), anti‐centromere (ACA; Antibodies Incorporated, 15–234, 1:100), anti‐SMC3 (Abcam, ab128919, 1:100), anti‐SMC4 (Novus Biologicals, NBP1‐86635, 1:100) and anti‐CENP‐A (Cell Signaling, #2048, 1:250). Alexa Fluor 488‐conjugated anti‐mouse (Jackson ImmunoResearch, 115‐545‐144, 1:500), Alexa Fluor 488‐conjugated anti‐rabbit (Jackson ImmunoResearch, 115‐545‐144 1:500), Alexa Fluor 594‐conjugated anti‐rabbit (Jackson ImmunoResearch, 111‐585‐144, 1:500) and Rhodamine (TRITC) ‐conjugated anti‐human (Jackson ImmunoResearch, 109‐025‐088, 1:100) were used as secondary antibodies.

### 2.5 Chromosome spreading

Oocytes were exposed to Tyrode's solution for approximately 1 min to remove zona pellucida. After a brief recovery in fresh medium, oocytes were fixed in 1% PFA in PBS containing 0.15% Triton X‐100 and 3 mM dithiothreitol (DTT). The slides were dried in a humid chamber at 42°C for several hours and then blocked with 3% BSA in PBS for 1 h at room temperature. Oocytes were incubated overnight at 4°C with primary antibodies and then for 2 h at room temperature with secondary antibodies. Chromosomes were stained with DAPI and examined using an LSM 900 laser scanning confocal microscope.

### 2.6 TUNEL assay

The TUNEL assay was performed as described previously.^18^ Briefly, after preparing chromosome spreads as described above, the oocytes were incubated with fluorescent‐conjugated terminal deoxynucleotide transferase dUTP for 2 h at 37°C using an In Situ Cell Death Detection kit (Roche) according to the manufacturer's instructions. After counterstaining with DAPI, the oocytes were mounted on glass slides and observed using an LSM 900 laser scanning confocal microscope.

### 2.7 Comet assay

The comet assay was performed using an Alkaline CometAssay kit (Trevigen) according to the manufacturer's instructions. Briefly, oocytes were mixed with melted agarose, placed on comet slides and subjected to electrophoresis. The comet signals were visualized by staining with SYBR Green (Invitrogen) and images were captured with a confocal microscope.

### 2.8 Quantification of fluorescence intensity

All images were acquired at pixel dimensions of 1024 × 1024 and are shown as the maximum intensity of the Z‐projections using an LSM 900 laser scanning confocal microscope (Zeiss). For the measurement of immunofluorescence intensity, images were captured with the same laser power and the mean intensity of the fluorescence signals was measured and normalized to the mean DAPI signal intensity. The data were analysed using ZEN 3.4 Blue (Zeiss) and ImageJ software (National Institutes of Health) under the same processing parameters.

### 2.9 Statistical analysis

GraphPad Prism 9.0 (GraphPad Software Inc.) was used for statistical analysis. Data are presented as mean ± *SEM* of at least three independent experiments unless stated otherwise. The Student's *t*‐test or one‐way ANOVA with Tukey's post hoc test was used to evaluate group differences. A value of *p* < 0.05 was considered to be statistically significant.

## 3 RESULTS

### 3.1 NHEJ is critical for the meiotic maturation of oocytes with DNA damage

It is generally considered that meiotic DSBs are repaired preferentially by HR.^12^ However, a recent study has shown that MII oocytes repair DSBs using NHEJ.^13^ Therefore, we initially determined the preferred repair pathway used by oocytes at different stages of meiotic maturation. To this end, we used specific inhibitors, B02 and SCR7. While B02 is a specific inhibitor of Rad51 and blocks HR repair, SCR7 inhibits DNA Ligase IV, which is responsible for the repair of DSBs via the NHEJ repair.^19^, ^20^ After treating with etoposide (ETP) for 30 mins, GV or MII oocytes were allowed to recover from DNA damage for 1 h in the presence of B02 or SCR7, and DSB levels were assessed by measuring γ‐H2AX levels. We found that B02 and SCR7 treatments increased γ‐H2AX levels in GV and MII oocytes, respectively (Figure S1). These results suggest that HR is the preferred DSB repair pathway during GV arrest, whereas NHEJ is preferred at the MII stage, implying a transition of DSB repair pathways from HR to NHEJ during meiotic maturation.

**FIGURE 1:**
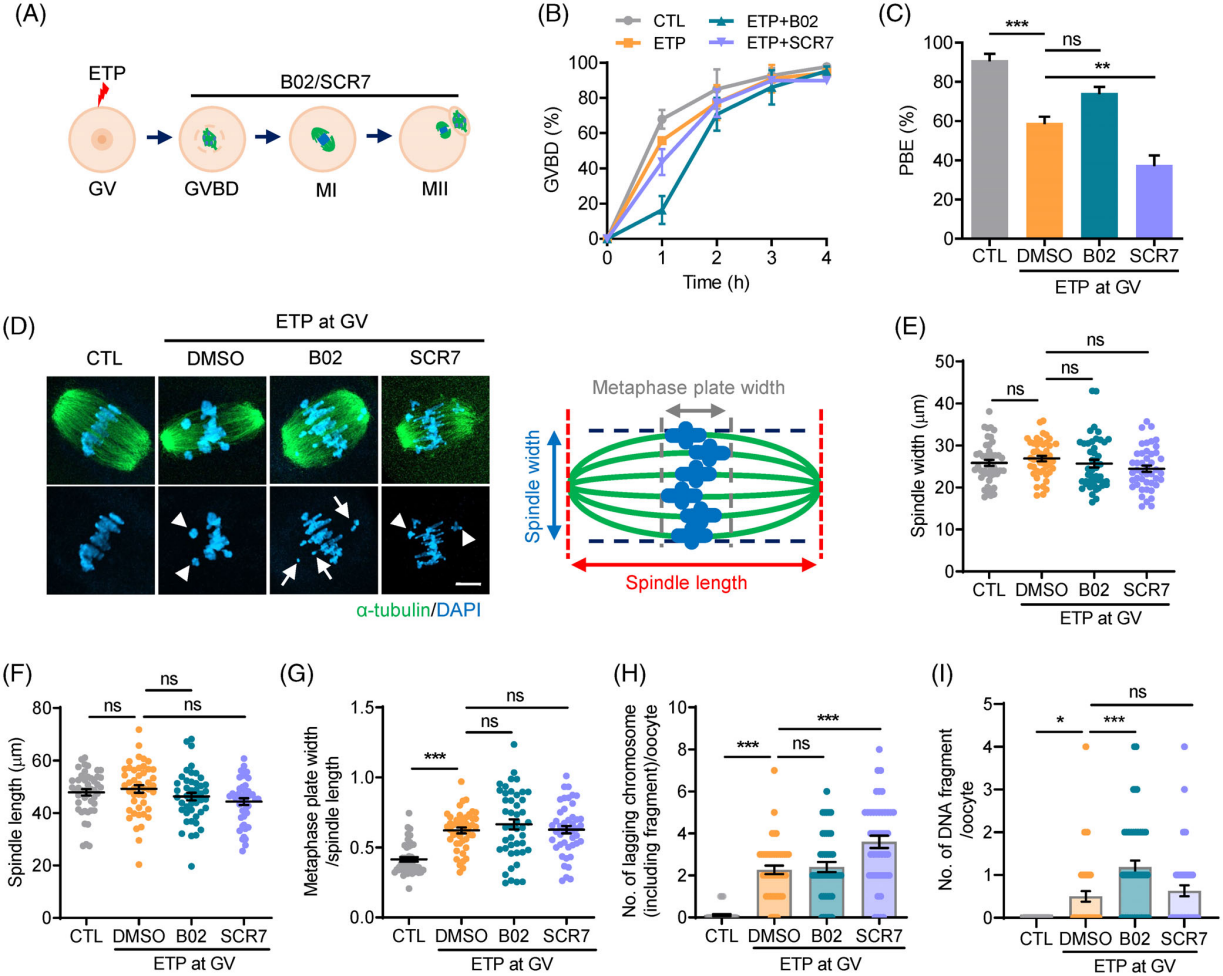
Effects of HR and NHEJ inhibition during the meiotic maturation of oocytes with DNA damage. (A) Experimental scheme of HR and NHEJ inhibition during meiotic maturation. After treating with etoposide (ETP) for 30 mins, oocytes were matured for 16 h with B02 or SCR7 to inhibit HR or NHEJ, respectively. (B,C) GVBD and PBE rates of oocytes. (D) Representative images of spindles and chromosomes in oocytes at the MI stage (8 h after IBMX release). Arrows and arrowheads indicate fragmented DNAs and lagging chromosomes, respectively. Scale bar, 10 μm. A schematic diagram of the meiotic spindle and chromosomes representing the spindle length and width and metaphase plate width is shown to the right of the images. (E,F) Quantification of spindle width and length. (G) Ratio of metaphase width to spindle length. (H,I) Number of lagging chromosomes and DNA fragments per oocyte. Data in the graphs are presented as mean ± *SEM* from three independent experiments. **p < 0.001, ***p < 0.0001, ns, not significant

To investigate the role of each DSB repair pathway during meiotic maturation, we matured GV oocytes pretreated with ETP for 30 mins in the presence of B02 or SCR7 (Figure 1A). Consistent with previous reports that oocytes with DNA damage do not show a G2/M checkpoint arrest,^7^ oocytes treated with ETP underwent GVBD comparable to the control. Moreover, neither B02 nor SCR7 treatment affected GVBD (Figure 1B). However, at 16 h after IBMX release, the polar body extrusion (PBE) rate significantly decreased after ETP treatment in line with previous studies that DNA damage induced MI arrest in oocytes.^9^, ^10^ Notably, the decrease in PBE rate was more pronounced by SCR7 treatment but was not affected by B02 treatment (Figure 1C). To further investigate the impact of inhibiting DSB repair pathways during meiotic maturation, we examined the spindle and chromosome organizations in oocytes at MI stages after treating with B02 or SCR7 during meiotic maturation. Immunostaining analyses revealed that ETP treatment increased the incidence of chromosome misalignment but did not affect overall spindle organization (Figure 1D–H). This is consistent with previous reports that oocytes with DNA damage resume meiosis and form normal bipolar spindles during meiotic maturation.^9^ Although SCR7 or B02 alone had little effect on the meiotic maturation of intact oocytes displaying normal spindle and chromosome organization (Figure S2), we found that SCR7 treatment during meiotic maturation in oocytes exposed to ETP significantly increased the number of lagging chromosomes. In contrast, B02 treatment in oocytes with DNA damage did not further increase the lagging chromosomes induced by ETP treatment, but slightly increased the number of DNA fragments (Figure 1D–I). Therefore, our results not only suggest that NHEJ is critical for the meiotic maturation of oocytes with DNA damage, but implies that HR and NHEJ have distinct roles during meiotic maturation.

### 3.2 Inhibition of HR or NHEJ increases DNA damage levels in oocytes with DNA damage during meiotic maturation

**FIGURE 2:**
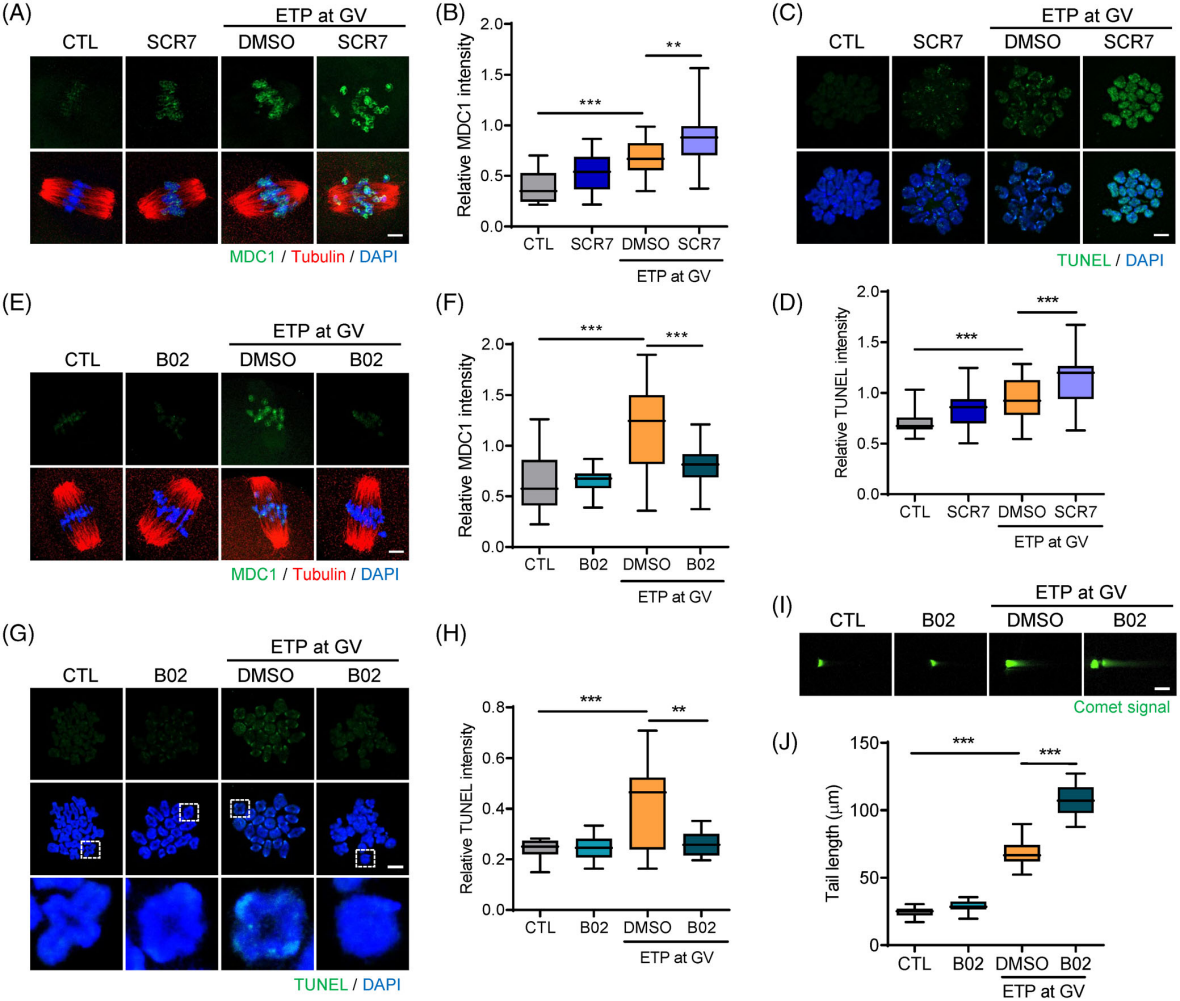
Inhibition of HR or NHEJ during meiotic maturation increases DNA damage levels in oocytes. GV oocytes exposed to ETP were matured with either SCR7 or B02. After 8 h of culturing, oocytes at the MI stage were subjected to immunostaining, TUNEL analysis, or comet assay. (A,E) Representative images showing MDC1 levels. Scale bar, 10 μm. (C,G) Representative images of chromosome spreads showing TUNEL signals. Individual chromosomes in the boxed region were shown at the bottom. Scale bar, 10 μm. (B,D,F,H) Quantification of MDC1 and TUNEL intensities. Data in the graphs are presented as the mean ± SEM from three independent experiments. **p < 0.001, ***p < 0.0001. (I) Representative images showing comet assay. Scale bar, 20 μm. (J) Quantification of tail length of comet. Data in the graphs are presented as the mean ± *SEM* from three independent experiments. ***p < 0.0001

Because SCR7 treatment disturbed the meiotic progression of oocytes with DNA damage, we asked whether this arrest is associated with the accumulation of DNA damage caused by NHEJ inhibition. To this end, we examined levels of MDC1, one of the most sensitive DSB markers.^18^, ^21^ While basal levels of chromosomal MDC1 signal were detectable in intact control oocytes, ETP‐treated oocytes exhibited increased chromosomal MDC1 levels (Figure 2A,B). Notably, the increase in MDC1 levels by ETP was more pronounced after SCR7 treatment during meiotic maturation. Moreover, TUNEL analysis showed that the levels of DNA damage induced by ETP were elevated by SCR7 treatment during meiotic maturation (Figure 2C,D). Unexpectedly, however, B02 treatment decreased the levels of MDC1 and TUNEL signals (Figure 2E–H). Because DNA‐damaged oocytes did not display the typical shape of bivalent chromosomes after B02 treatment (Figure 2G), we thought that the decrease in MDC1 and TUNEL signals are likely associated with abnormalities in chromosome structure. To clarify this, we performed a comet assay and found that a substantial level of DNA damage remained after B02 treatment (Figure 2I,J). Importantly, the tail length of comet was found to increase more than that of the ETP‐treated control oocytes. Therefore, our results suggest that HR and NHEJ play an important role in DSB repair in a distinct manner during meiotic maturation.

### 3.3 Inhibition of NHEJ increases the incidence of SAC‐mediated MI arrest in oocytes with DNA damage

**FIGURE 3:**
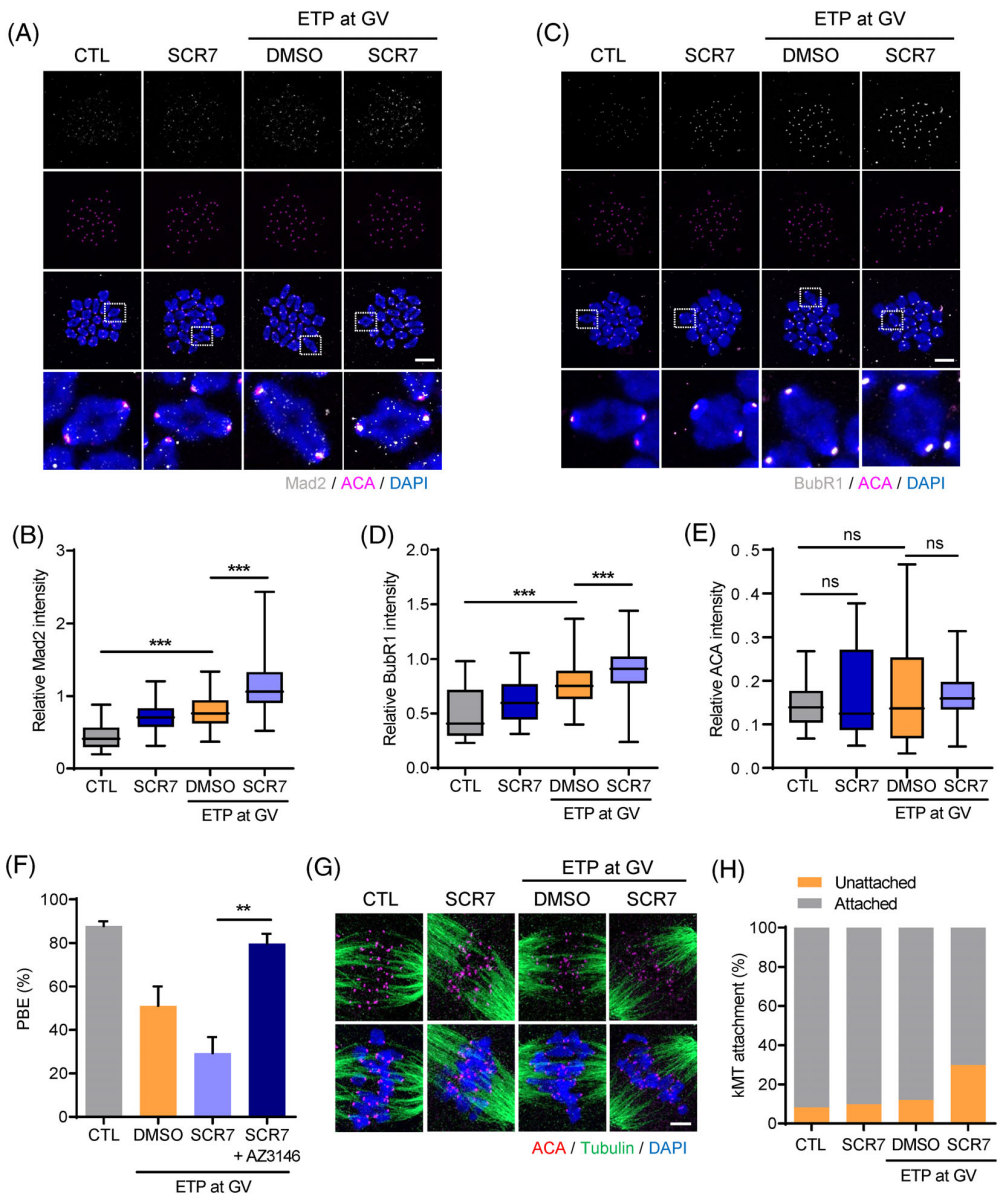
Inhibition of NHEJ during meiotic maturation increases the incidence of MI arrest by activating SAC and aberrant kMT attachments. (A–D) GV oocytes exposed to ETP were matured with SCR7. After 8 h of culturing, oocytes at the MI stage were subjected to chromosome spreading. (A,C) Representative chromosome spreads of MI oocytes stained with Mad2 and BubR1 antibodies. Scale bar, 10 μm. (B,D) Quantification of Mad2 and BubR1 intensities normalized to ACA signals. (E) Quantification of ACA intensities from the experiments illustrated in panel A and C. (F) PBE rates of oocytes after AZ3146 treatment during meiotic maturation. Data in the graphs are presented as mean ± *SEM* from three independent experiments. **p < 0.001, ***p < 0.0001, ns, not significant. (G) Representative images of kMT attachments in MI oocytes. Scale bar, 10 μm. (H) Quantification of kMT attachments

It has been well established that oocytes with DNA damage induce SAC‐mediated MI arrest.^9^, ^10^ Therefore, we assumed that the increased MI arrest caused by NHEJ inhibition during the meiotic maturation of oocytes with DNA damage is associated with SAC activation. To investigate this, we determined Mad2 and BubR1 levels after SCR7 treatment during meiotic maturation. Compared to control oocytes, DNA‐damaged oocytes exhibited elevated levels of Mad2 and BubR1 (Figure 3A–D), consistent with previous reports that DNA damage induced SAC‐mediated MI arrest.^9^, ^10^ Importantly, the increase in Mad2 and BubR1 levels was more pronounced without affecting centromere integrity when DNA‐damaged oocytes were treated with SCR7 during meiotic maturation (Figure 3A–E), supporting the association between the observed MI arrest after SCR7 treatment and SAC activation. Indeed, MI arrest caused by SCR7 treatment was rescued by SAC inactivation using Mps1 inhibitor AZ3146 (Figure 3F). Moreover, we found that kMT attachments were not impaired after ETP treatment (Figure 3G,H), which is consistent with previous reports that ETP‐induced SAC arrest is not associated with aberrant kMT attachment. However, SCR7 treatment during meiotic maturation slightly increased impaired kMT attachments (Figure 3G,H). Taken together, our results suggest that, in oocytes with DNA damage, NHEJ inhibition during meiotic maturation increases DNA damage levels, which in turn increases SAC‐mediated MI arrest with aberrant kMT attachments.

### 3.4 Inhibition of HR bypasses SAC‐mediated MI arrest in oocytes with DNA damage

**FIGURE 4:**
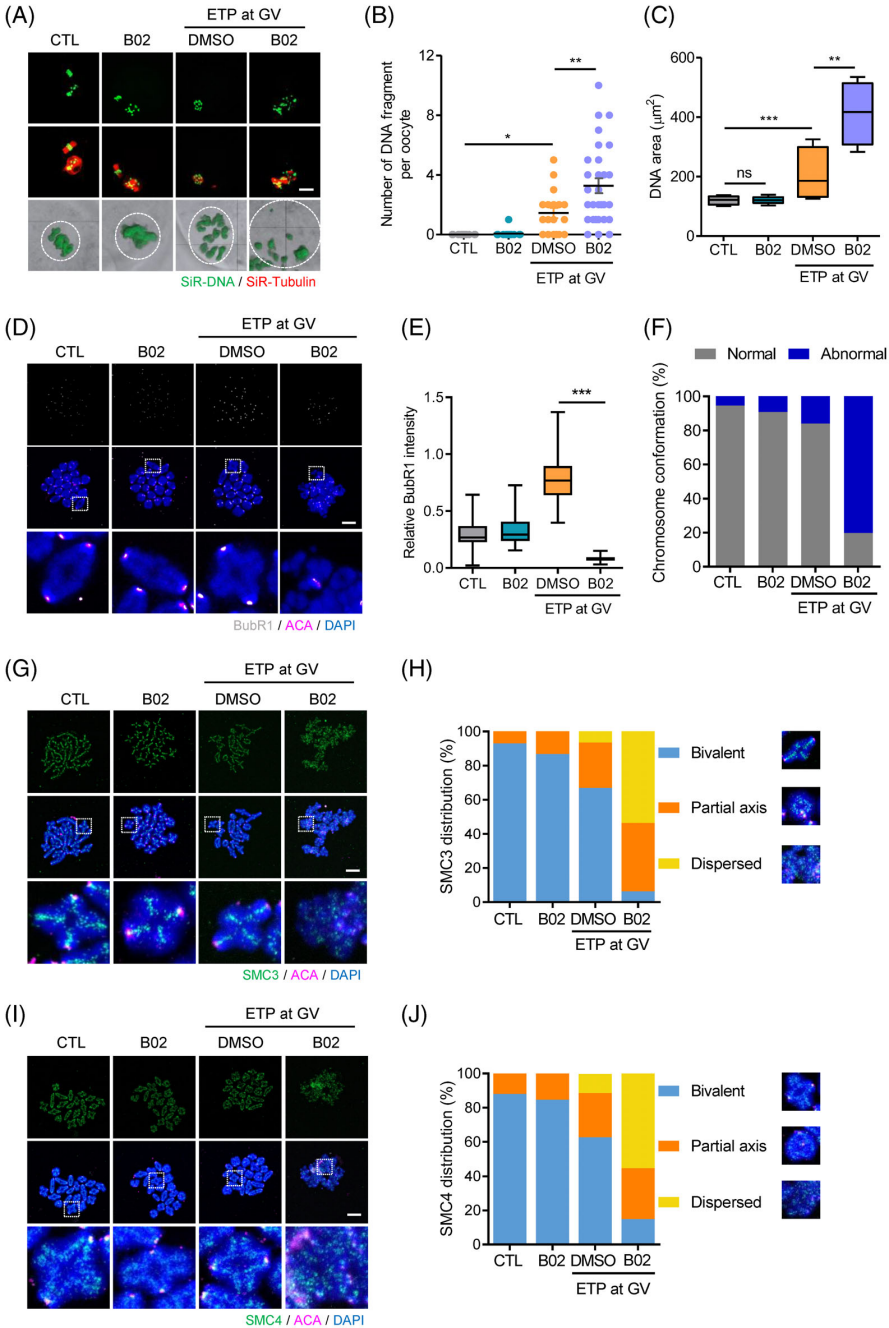
Inhibition of HR during meiotic maturation impairs chromosome architecture. (A) Representative images of spindles and chromosomes in MII oocytes. Scale bar, 10 μm. The 3D‐reconstructed images of chromosomes (green) are shown in the bottom panel. Chromosome area in oocytes is marked with dotted circles. Corresponding 3D‐reconstructed spindle and chromosomes are provided in the supplementary materials ([Link], [Link]). (B) The number of DNA fragments per oocyte. (C) Quantification of chromosome area in oocytes. Data in the graphs are presented as mean ± *SEM* from three independent experiments. *p < 0.05, **p < 0.001, ***p < 0.0001, ns, not significant. (D) Representative chromosome spreads of MI oocytes stained with BubR1 and ACA. The bottom panels show representative, individual chromosomes in the boxed region. Scale bar, 10 μm. (E) Quantification of BubR1 intensity normalized to DAPI signals. ***p < 0.0001. (F) Quantification of chromosome configuration. (G,I) Representative images of chromosome spreads showing SMC3 and SMC4 distribution. The bottom panels show representative, individual chromosomes in the boxed region. Scale bar, 10 μm. (H,J) Quantification of SMC3 and SMC4 distribution on bivalent chromosomes. The distribution of SMC3 or SMC4 on individual chromosomes was classified as bivalent, partial axis, or dispersed.

Although B02 treatment had little effect on meiotic progression in oocytes with DNA damage, the impaired chromosome structure led us to investigate the possible role of HR during meiotic maturation in response to DNA damage. Thus, we examined the chromosome configuration of MII oocytes matured in the presence of B02. Surprisingly, we found that chromosomes were highly disorganized and fragmented after B02 treatment during the meiotic maturation of oocytes with DNA damage (Figure 4A–C; see Supplementary [Link], [Link] for full 3D‐reconstructed oocytes showing spindle and chromosomes). Because oocytes progressed to MII stage despite the presence of fragmented DNA, we wondered how oocytes bypassed SAC‐mediated MI arrest. To investigate this, we examined BubR1 levels on MI chromosomes. Interestingly, the elevated BubR1 levels induced by ETP treatment dramatically decreased after B02 treatment during meiotic maturation (Figure 4D,E). More interestingly, B02 treatment disrupted the traditional cruciform structure of bivalent chromosomes in MI oocytes (Figure 4D,F). This result led us to investigate whether reduced BubR1 levels after B02 treatment are likely due to disrupted chromosome structures rather than SAC inactivation. Because condensins and cohesins are essential to maintain metaphase chromosomal structure, we determined the distribution of condensins and cohesins on bivalent chromosomes. While ETP treatment did not change the traditional distribution of SMC3 (cohesin subunit) along the axis between sister chromatids, B02 treatment after ETP exposure severely impaired SMC3 distribution on bivalent chromosomes (Figure 4G,H). Similar to SMC3, the distribution of SMC4 (condensin subunit) was also impaired by B02 treatment after ETP exposure (Figure 4I,J). Therefore, our results suggest not only that the bypass of SAC‐mediated arrest after B02 treatment is likely associated with impaired chromosome structures but also that the HR is required to maintain the structure of bivalent chromosomes during meiotic maturation in DNA‐damaged oocytes.

### 3.5 HR is essential to maintaining centromere integrity in oocytes with DNA damage

**FIGURE 5:**
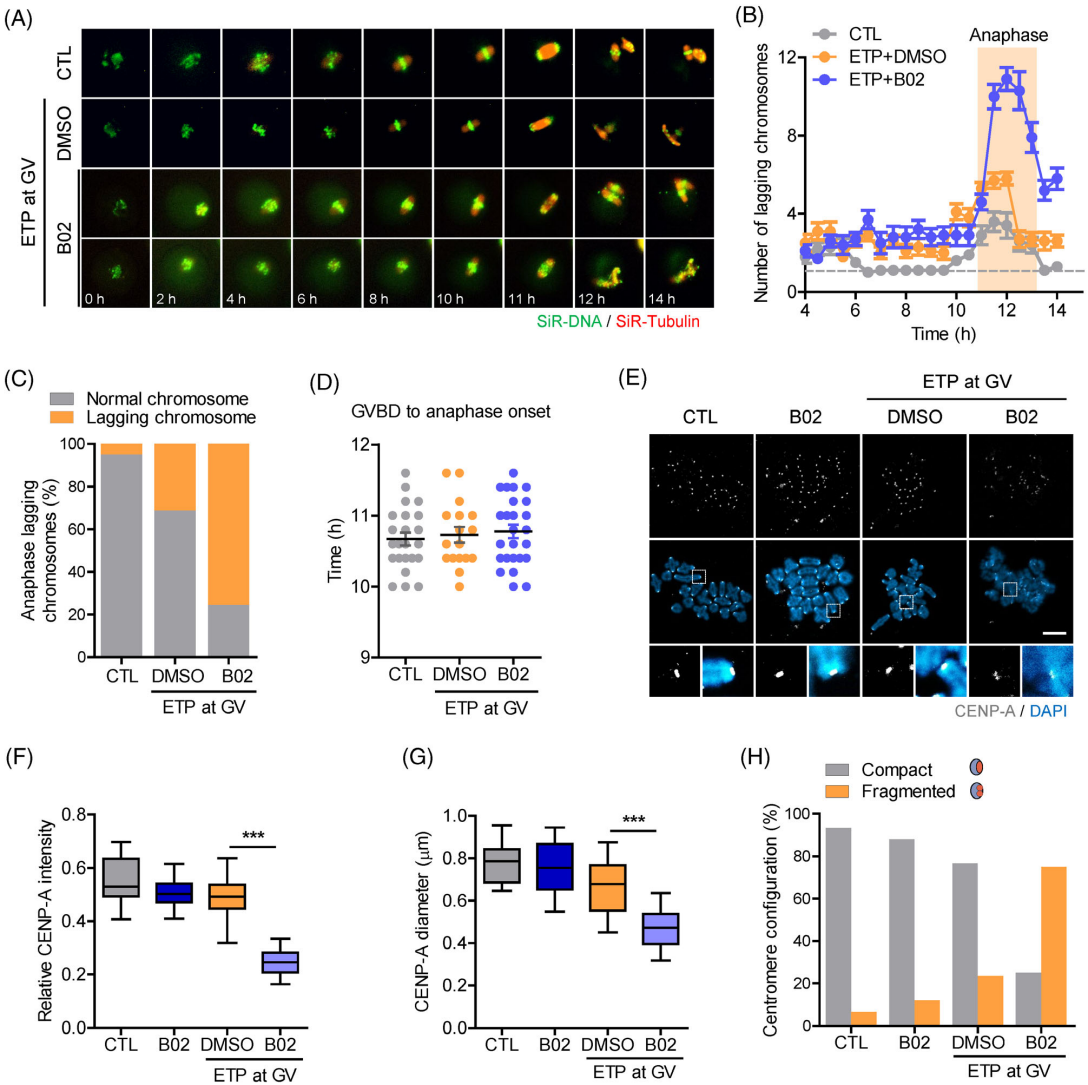
Inhibition of HR during meiotic maturation decreases centromere integrity and causes DNA fragmentation. (A) Representative time‐lapse images of spindles and chromosomes during meiotic maturation. Corresponding movies are provided in the supplementary materials ([Link], [Link]). Scale bar, 10 μm. (B) The mean number of lagging chromosomes per oocyte during meiotic maturation. The orange box highlights anaphase I. (C) Quantification of lagging chromosomes during anaphase I. (D) Average time from GVBD to anaphase onset in oocytes. (E) Representative chromosome spreads of MI oocytes stained with CENP‐A. The bottom panels show centromeric regions of individual chromosomes in the boxed region. Scale bar, 10 μm. (F) Quantification of CENP‐A intensity in individual chromosomes. (G) Quantification of CENP‐A diameter in individual chromosomes. ***p < 0.0001. (H) Quantification of centromere configuration based on CENP‐A signals at bivalent chromosomes.

Because DNA fragments were frequently observed after B02 treatment in oocytes with DNA damage, we wanted to monitor chromosome behaviour during meiotic maturation. To this end, we performed time‐lapse imaging of oocytes after labelling the chromosomes and spindle microtubules with SiR‐DNA and SiR‐tubulin, respectively. In control oocytes, chromosomes were aligned in the middle of the spindle with the establishment of a metaphase plate until metaphase and then equally segregated at anaphase. In ETP‐treated oocytes, however, although most chromosomes were well‐aligned at metaphase, some lagging chromosomes appeared during anaphase. Strikingly, the number of lagging chromosomes dramatically increased after B02 treatment during anaphase I (Figure 5A–C; see Supplementary [Link], [Link]). This is not due to the acceleration of meiosis I, as the timing of PBE after GVBD was not different between groups (Figure 5D). Therefore, our results suggest that DNA fragmentation mainly occurs during chromosome segregation at anaphase I in oocytes with DNA damage when HR is blocked during meiotic maturation.

Given that the centromere is essential for kinetochore assembly and subsequent recruitment of SAC proteins, in addition to being required to maintain chromosome structure,^22^ we reasoned that the observed bypass of SAC and DNA fragmentation after B02 treatment were likely due to reduced integrity of the centromere, which in turn impairs kinetochore assembly and the recruitment of SAC proteins, as well as chromosome fragmentation. To investigate this, we examined fragmented DNA and its association with CENP‐A. We found that CENP‐A intensity dramatically decreased after B02 treatment (Figure 5E,F). Moreover, decompaction and fragmentation of CENP‐A were frequently observed after B02 treatment (Figure 5E,G,H). This result implies that many DNA fragments observed after B02 treatment were derived from centromere fragmentation. Collectively, our results suggest that HR is required to maintain centromere integrity in response to DNA damage during meiotic maturation in oocytes.

## 4 DISCUSSION

**FIGURE 6:**
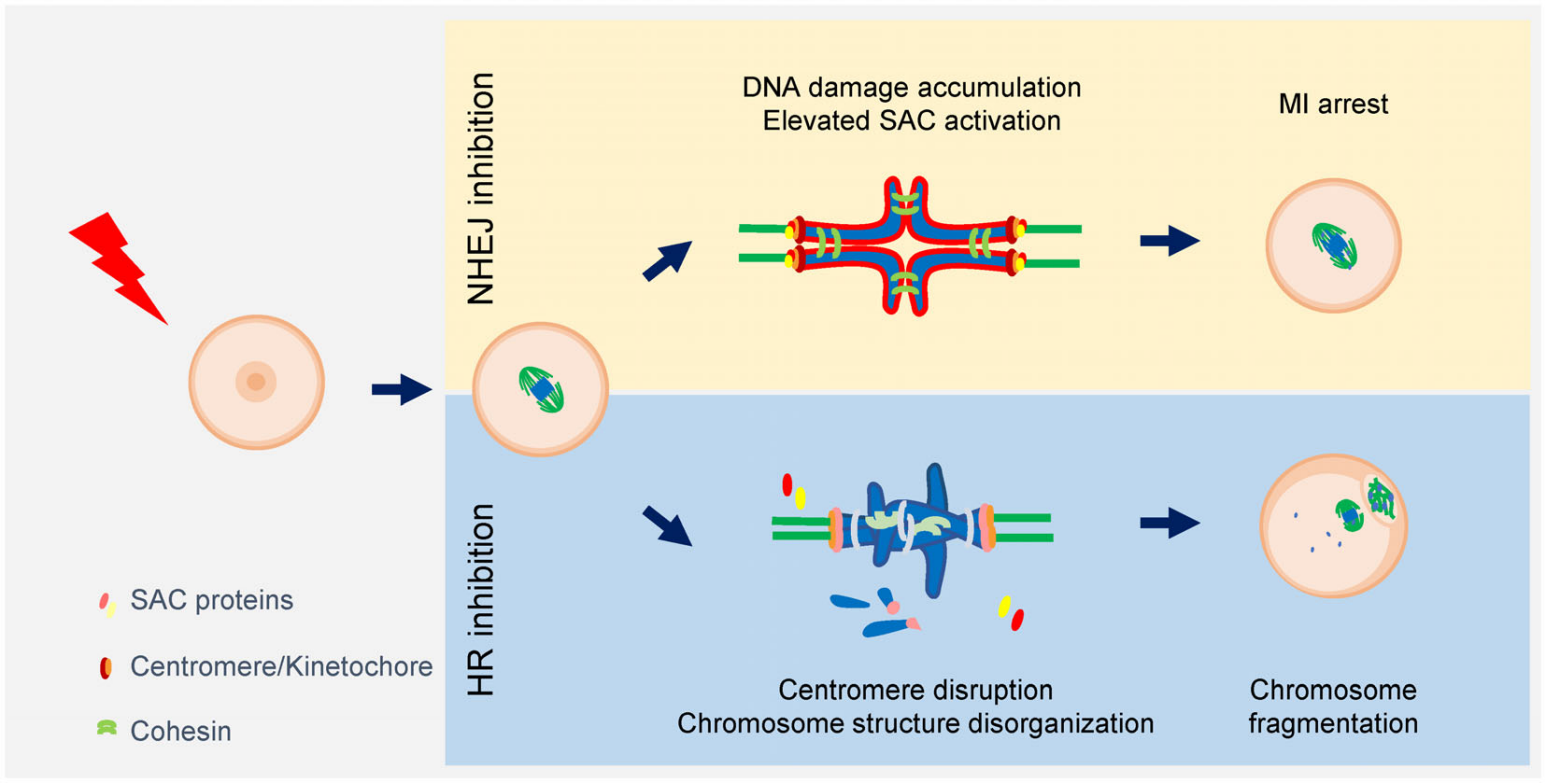
Scheme summarizing the distinct roles of HR and NHEJ during meiotic maturation in oocytes with DNA damage. Inhibition of NHEJ impairs meiotic maturation in oocytes with DNA damage by increasing DNA damage levels and, subsequently, the incidence of SAC‐mediated MI arrest. In contrast, inhibition of HR decreases centromere integrity, which in turn causes DNA fragmentation during chromosome segregation

When cells encounter DSBs during interphase, they can halt cell cycle progression and repair DSBs before entering mitosis. During mitosis, however, cells do not mount cell cycle arrest and DNA repair. While the compact architecture of mitotic chromatin is thought to confer a protective role, unrepaired DSBs may cause serious chromosomal defects that can lead to cancers. In contrast to mitotic cells, DSBs are produced by endonuclease activity between two homologous chromosomes and then repaired by HR to generate genetic diversity in offspring during meiosis, indicating that DSBs in chromosomes can be repaired during oocyte meiosis. Although mounting evidence suggests that oocytes in meiosis have a capacity to repair DSBs, the role of each repair pathway during meiotic maturation remains largely unknown. In this study, we found that oocytes selectively utilize NHEJ and HR to repair DSBs during meiotic maturation (Figure 6).

It has been well documented that DNA damage in oocytes does not activate the G2/M checkpoint but does induce SAC‐mediated MI arrest during meiotic maturation.^7^, ^8^, ^9^, ^10^ Consistent with this, our results showed that oocytes exposed to ETP at the GV stage underwent GVBD and were arrested at the MI stage. However, the incidence of MI arrest induced by ETP treatment at the GV stage was elevated by NHEJ inhibition during meiotic maturation. Because ETP caused the MI arrest in a dose‐dependent manner,^9^ we assumed that the increase in MI arrest by NHEJ inhibition was associated with increased DNA damage in oocytes during meiotic maturation. Indeed, we observed that the MI‐arrested oocytes exhibited higher levels of DNA damage and accompanied severe chromosomal abnormality after NHEJ inhibition. Moreover, this MI arrest was rescued by SAC inactivation. However, we found that NHEJ inhibition partially impaired kMT attachments, which is not consistent with previous reports that DNA damage‐induced SAC arrest in oocytes is not associated with aberrant kMT attachments.^11^ Given that TUNEL signals significantly increased after NHEJ inhibition, it is probably due to the accumulation of DNA damage beyond the repair capacity of oocytes after NHEJ inhibition. Therefore, our findings suggest that the inhibition of NHEJ impairs the repair capacity of oocytes and thereby increases DNA damage levels after GVBD, which in turn activates SAC and causes MI arrest, suggesting that oocytes are able to repair DSBs using NHEJ during meiotic maturation. Consistent with this notion, UV irradiation or treatment with mutagenic drugs has been shown to induce unscheduled DNA synthesis in GV, MI and MII oocytes,^23^ supporting the notion that oocytes can repair DNA damage during meiotic maturation. Given that HR is a dominant repair pathway at the GV stage, our data also suggest that oocytes switch DSB repair pathways from HR to NHEJ after GVBD. Therefore, it is possible that, in intact oocytes, NHEJ inhibition alone during meiotic maturation could induce the accumulation of DNA damage, considering the nature of in vitro culture conditions that may increase oxidative stress and environmental damage, including DNA damage.^15^, ^24^, ^25^ In this regard, the observed decrease in PBE rate after SCR7 treatment during meiotic maturation in intact oocytes is likely associated with accumulated DNA damage during in vitro maturation. Given the intricacies in DSB repair between HR and NHEJ during cell cycle progression, the compensatory role of HR when NHEJ is suppressed or vice versa is also considerable. Further studies are required to clarify the crosstalk between HR and NHEJ during meiotic maturation in mouse oocytes.

We observed that oocytes successfully matured to the MII stage, extruding polar bodies, when HR was inhibited during meiotic maturation in oocytes with DNA damage. However, a closer look at the molecular levels revealed that, despite completing meiotic progression, the chromosomes in these oocytes were highly disorganized with a number of DNA fragments. Moreover, many of the DNA fragments contained CENP‐A domains and were generated during chromosome segregation at anaphase I. These data suggest that HR is required to maintain centromere integrity during oocyte meiosis. In contrast to NHEJ, HR requires a sequence similar or identical to the broken DNA as a template for DSB repair. Given that centromeres consist of tandem arrays of highly repetitive sequences, DSBs in the centromeric regions might be preferentially repaired by HR.^26^ Indeed, a recent study demonstrated that the HR machinery was recruited to DSBs at centromeres even in the absence of sister chromatids at G1 phase in somatic cells.^27^ Moreover, pericentromeric regions enrich condensin and cohesin complexes, which are structural subunits essential to establish the organization of the metaphase chromosome structure.^28^, ^29^, ^30^, ^31^ Thus, it is likely that the observed disorganization and fragmentation of bivalent chromosomes after inhibiting HR during meiotic maturation are associated with the loss of centromere integrity and impaired distribution of condensin and cohesin. The failure to preserve centromere integrity further impairs kinetochore assembly and SAC activation, resulting in the bypass of SAC arrest. Because p‐MDC1 is localized at the spindle pole during oocyte meiosis,^32^ the decrease in MDC1 and TUNEL signals after B02 treatment during meiotic maturation of oocytes with DNA damage is likely associated with impaired centromere and chromosome architecture. Moreover, weakened centromeres tend to be fragmented due to the increased centromere tension generated by the pulling force of spindle microtubules during chromosome segregation. Given that the centromere is an essential chromosomal element that enables faithful chromosome segregation during cell division and is fragile and prone to rearrangement, it is of particular importance to preserve centromere integrity during cell division. In this respect, our data suggest that HR acts as a guardian of centromeres during meiotic maturation in mouse oocytes.

Collectively, in response to DNA damage, NHEJ is crucial for successful meiotic progression to the MII stage by mediating the SAC, whereas HR is essential to maintaining centromere integrity during meiotic maturation. This finding is important as proper DNA damage repair ensures chromosome integrity, which then maintains oocyte quality and prevents future consequences caused by abnormal chromosomes.

## AUTHOR CONTRIBUTIONS

CL, JL and JSO conceived and designed the experiments. CL and JL performed all experiments. CL, JL and JSO analysed and interpreted the data. JSO supervised the study. JSO wrote the manuscript.

## FUNDING INFORMATION

This work was supported by the Basic Science Research Programme through the National Research Foundation of Korea (NRF) funded by the Ministry of Education (NRF‐2017R1A6A1A03015642 and NRF‐2019R1I1A2A01041413).

## CONFLICT OF INTEREST

The authors declare that they have no competing interests.

## Supporting information


**Movie S1.** 3D‐reconstructed images of chromosomes in Figure 4A (CTL).

**Movie S2.** 3D‐reconstructed images of chromosomes in Figure 4A (B02).

**Movie S3.** 3D‐reconstructed images of chromosomes in Figure 4A (ETP at GV + DMSO).

**Movie S4.** 3D‐reconstructed images of chromosomes in Figure 4A (ETP at GV + B02).

**Movie S5.** Time lapse images of spindles and chromosomes during meiotic maturation in Figure 5A (CTL).

**Movie S6.** Time lapse images of spindles and chromosomes during meiotic maturation in Figure 5A (ETP at GV + DMSO).

**Movie S7.** Time lapse images of spindles and chromosomes during meiotic maturation in Figure 5A (ETP at GV + B02; upper panel).

**Movie S8.** Time lapse images of spindles and chromosomes during meiotic maturation in Figure 5A (ETP at GV + B02; bottom panel).

**Figure S1.** Preferential DSB repair pathways for GV and MII oocytes. After exposure to ETP for 30 mins, GV or MII oocytes were allowed to recover in ETP‐free media for 1 h and then subjected to immunostaining analysis with γ‐H2AX. (A,C) Representative images of GV (A) or MII (C) oocytes. (B,D) Quantification of γ‐H2AX intensity. Scale bar, 10 μm. ns, not significant.
**Figure S2.** Effects of HR or NHEJ inhibition during meiotic maturation. Intact oocytes (not exposed to ETP) were matured for 16 h with B02 or SCR7. (A,B) GVBD and PBE rates of oocytes. (C) Representative images of spindles and chromosomes in MI and MII oocytes. Scale bar, 10 μm. (D,E) Quantification of spindle width and length of MI oocytes. (F) Ratio of metaphase plate width to spindle length of MI oocytes. (G,H) Quantification of spindle width and length of MII oocytes. Data in graphs are presented as mean ± *SEM* from three independent experiments. *p < 0.05, ns, not significant.

## Data Availability

All data needed to evaluate the conclusions in the paper are presented in the paper and/or the Supplementary Materials. Additional data related to this paper can be requested from the authors.
