# Supplementary material for: Selective utilization of non‐homologous end‐joining and homologous recombination for DNA repair during meiotic maturation in mouse oocytes
Source: Cell Prolif. 2022 Dec 23;56(4):e13384. doi: 10.1111/cpr.13384 (PMC10068936; doi:10.1111/cpr.13384)
Supplement: Supplementary file 9 — Figure S1. Preferential DSB repair pathways for GV and MII oocytes. After exposure to ETP for 30 mins, GV or MII oocytes were allowed to recover in ETP‐free media for 1 h and then subjected to immunostaining analysis with γ‐H2AX. (A,C) Representative images of GV (A) or MII (C) oocytes. (B,D) Quantification of γ‐H2AX intensity. Scale bar, 10 μm. ns, not significant. Figure S2. Effects of HR or NHEJ inhibition during meiotic maturation. Intact oocytes (not exposed to ETP) were matured for 16 h with B02 or SCR7. (A,B) GVBD and PBE rates of oocytes. (C) Representative images of spindles and chromosomes in MI and MII oocytes. Scale bar, 10 μm. (D,E) Quantification of spindle width and length of MI oocytes. (F) Ratio of metaphase plate width to spindle length of MI oocytes. (G,H) Quantification of spindle width and length of MII oocytes. Data in graphs are presented as mean ± SEM from three independent experiments. *p < 0.05, ns, not significant. [file CPR-56-e13384-s001.docx]

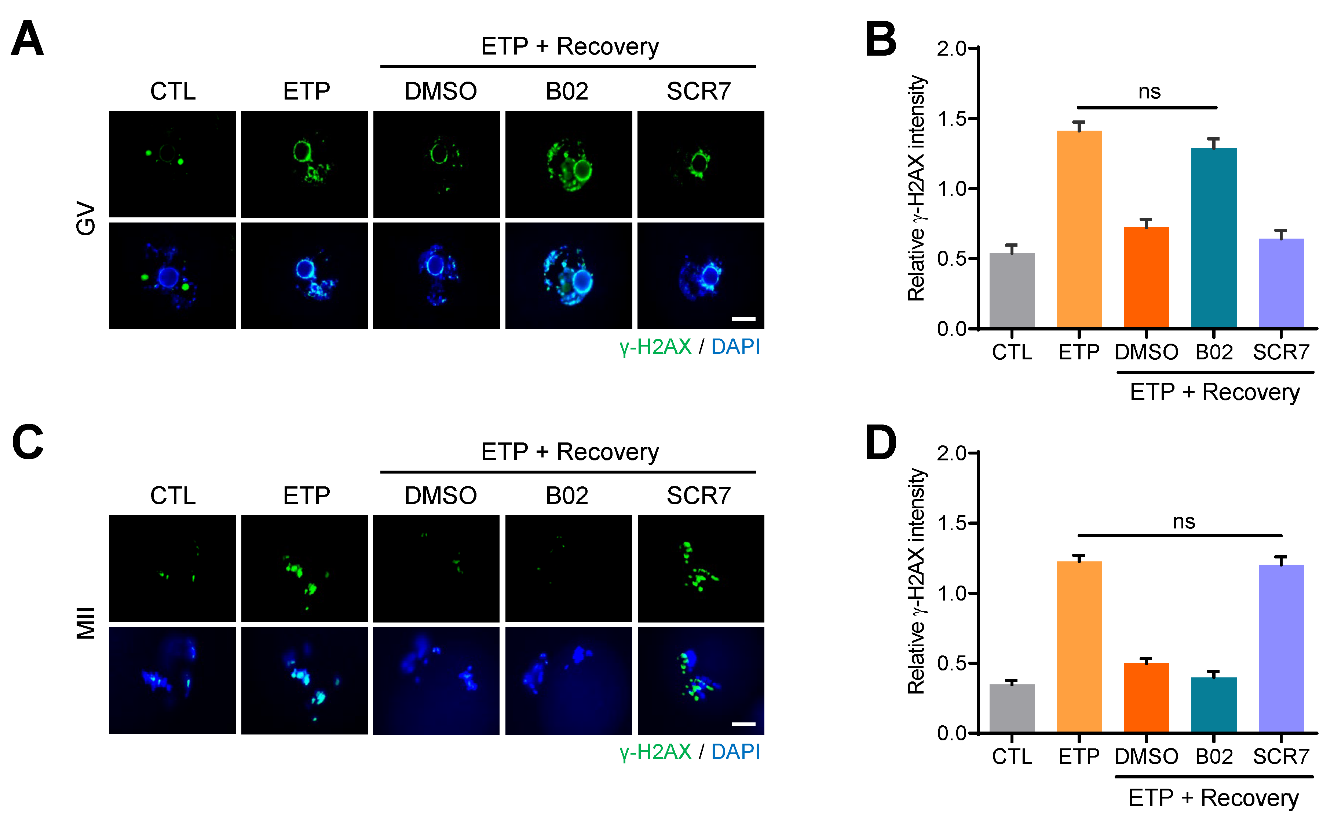


**Fig. S1. Preferential DSB repair pathways for GV and MII oocytes.** After exposure to ETP for 30 min, GV or MII oocytes were allowed to recover in ETP-free media for 1 h and then subjected to immunostaining analysis with γ-H2AX. (A, C) Representative images of GV (A) or MII (C) oocytes. (B, D) Quantification of γ-H2AX intensity. Scale bar, 10 μm. ns, not significant.


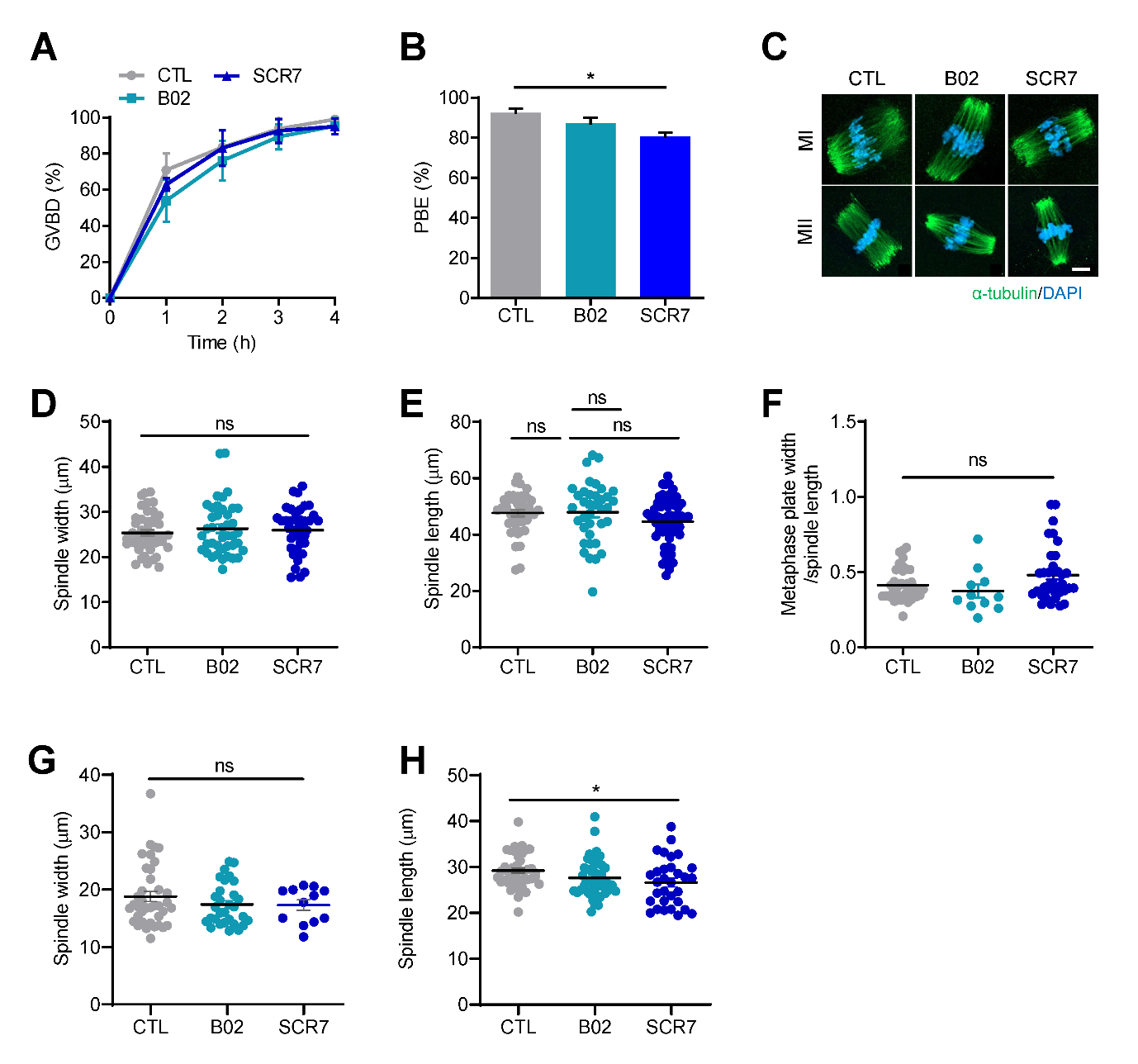


**Fig. S2. Effects of HR or NHEJ inhibition during meiotic maturation.** Intact oocytes (not exposed to ETP) were matured for 16 h with B02 or SCR7. (A, B) GVBD and PBE rates of oocytes. (C) Representative images of spindles and chromosomes in MI and MII oocytes. Scale bar, 10 μm. (D, E) Quantification of spindle width and length of MI oocytes. (F) Ratio of metaphase plate width to spindle length of MI oocytes. (G, H) Quantification of spindle width and length of MII oocytes. Data in graphs are presented as mean ± SEM from three independent experiments. *p<0.05, ns, not significant.

**Movie 1. 3D-reconstructed images of chromosomes in Fig. 4A (CTL).**

**Movie 2. 3D-reconstructed images of chromosomes in Fig. 4A (B02).**

**Movie 3. 3D-reconstructed images of chromosomes in Fig. 4A (ETP at GV + DMSO).**

**Movie 4. 3D-reconstructed images of chromosomes in Fig. 4A (ETP at GV + B02).**

**Movie 5. Time lapse images of spindles and chromosomes during meiotic maturation in Fig. 5A (CTL).**

**Movie 6. Time lapse images of spindles and chromosomes during meiotic maturation in Fig. 5A (ETP at GV + DMSO).**

**Movie 7. Time lapse images of spindles and chromosomes during meiotic maturation in Fig. 5A (ETP at GV + B02; upper panel).**

**Movie 6. Time lapse images of spindles and chromosomes during meiotic maturation in Fig. 5A (ETP at GV + B02; bottom panel).**
